# The Story of the Insane from Year to Year

**Published:** 1898-04-23

**Authors:** 


### THE STORY OF THE INSANE FROM YEAR TO YEAR.

### Tenth Series.

### THREE COUNTIES ASYLUM AT BALDOCK, HERTS.

Here the numbers were almost stationary during 1896, being 1,080 at the beginning of the year and 1,076 at the close of it. There were 199 first admissions, 46 readmissions, 119 recoveries, 7 discharged relieved, and 87 deaths. The recoveries stand at 48·5 per cent. on the admissions, and the deaths are 8·16 per cent. on the average number resident. Of the deaths 36 were caused by cerebral and spinal diseases, 14 by consumption, and 1 by pneumonia. Moral causes account for the insanity in 25 of the year's admissions, intemperance in drink for 20, and hereditary influence for 71. The asylum is again more than full, and contracts have been entered into with other asylums to receive 100 patients, but even this number does not give sufficient relief, and accommodation has to be found in Norfolk for 40 cases. The Commissioners recommend the appointment of another assistant medical officer, but the committee did not carry the recommendation into effect. The committee say that "Dr. Swain continues to devote his skill and abilities to the amelioration of the sad condition of the patients." The cost of maintenance was 8s. 4¼d. per head per week.

